# Drug eluting balloons for *de novo* coronary lesions – a systematic review and meta-analysis

**DOI:** 10.1186/1741-7015-11-123

**Published:** 2013-05-08

**Authors:** Georg M Fröhlich, Alexandra J Lansky, Dennis T Ko, Olga Archangelidi, Rodney De Palma, Adam Timmis, Pascal Meier

**Affiliations:** 1 The Heart Hospital, University College London Hospital, 16-18 Westmoreland Street, London, W1G 8PH, UK; 2 Division of Cardiology, Yale Medical School, 333 Cedar Street, New Haven, CT, 06510, USA; 3 Institute for Clinical Evaluative Sciences, 2075 Bayview Ave., Toronto, ON, M4N 3M5, Canada; 4 Department of Epidemiology and Public Health, University College London UCL, 1-19 Torrington Place, London, WC1E 6BT, UK; 5 Department of Cardiology, London Chest Hospital, Bethnal Green, London, E2 9JX, UK

**Keywords:** Coronary artery disease, Stents, Drug-eluting balloon, Percutaneous coronary intervention

## Abstract

**Background:**

The role of drug-eluting balloons (DEB) is unclear. Increasing evidence has shown a benefit for the treatment of in-stent restenosis. Its effect on *de novo* coronary lesions is more controversial. Several smaller randomized trials found conflicting results.

**Methods:**

This is a systematic review and meta-analysis of randomized controlled trials (RCT) evaluating the effect of local Paclitaxel delivery/drug eluting balloons (DEB) (+/− bare metal stent) compared to current standard therapy (stenting) to treat *de novo* coronary lesions. Data sources for RCT were identified through a literature search from 2005 through 28 December 2012. The main endpoints of interest were target lesion revascularization (TLR), major adverse cardiac events (MACE), binary in-segment restenosis, stent thrombosis (ST), myocardial infarction (MI), late lumen loss (LLL) and mortality. A random effects model was used to calculate the pooled relative risks (RR) with 95% confidence intervals.

**Results:**

Eight studies (11 subgroups) and a total of 1,706 patients were included in this analysis. Follow-up duration ranged from 6 to 12 months. Overall, DEB showed similar results to the comparator treatment. The relative risk (RR) for MACE was 0.95 (0.64 to 1.39); *P* = 0.776, for mortality it was 0.79 (0.30 to 2.11), *P* = 0.644, for stent thrombosis it was 1.45 (0.42 to 5.01), *P* = 0.560, for MI it was 1.26 (0.49 to 3.21), *P* = 0.629, for TLR it was 1.09 (0.71 to 1.68); *P* = 0.700 and for binary in-stent restenosis it was 0.96 (0.48 to 1.93), *P* = 0.918. Compared to bare metal stents (BMS), DEB showed a lower LLL (− 0.26 mm (−0.51 to 0.01)) and a trend towards a lower MACE risk (RR 0.66 (0.43 to 1.02)).

**Conclusion:**

Overall, drug-eluting balloons (+/− bare metal stent) are not superior to current standard therapies (BMS or drug eluting stent (DES)) in treating *de novo* coronary lesions. However, the performance of DEB seems to lie in between DES and BMS with a trend towards superiority over BMS alone. Therefore, DEB may be considered in patients with contraindications for DES. The heterogeneity between the included studies is a limitation of this meta-analysis; different drug-eluting balloons have been used.

## Background

Approximately 600,000 percutaneous coronary interventions (PCI) are performed in the United States each year [1]. Even though the frequency of restenosis after PCI and need for repeat revascularization has been reduced with the advent of drug eluting stents (DES), it still remains the main disadvantage compared to coronary bypass surgery [2]. Another Achilles heel of DES is the need for longer term dual antiplatelet therapy of usually 12 months’ duration compared to only one month for bare metal stents (BMS) [3,4]. Due to this limitation, there remains a proportion of about 20 to 30% of patients where bare metal stents are preferred [5]. Despite stent design improvement, drug-eluting and bare-metal stents both come with a worrying risk of stent thrombosis [6]. It would be optimal if PCI could be performed without leaving behind a permanent device. One option is the use of bioabsorbable scaffolds [7]. Another alternative includes the use of drug eluting balloons (DEB). DEB have been of proven benefit for the treatment of in-stent restenosis in several small randomized trials [8]. However, the use of DEB in the setting of *de novo* coronary lesions has only been addressed in a few smaller randomized trials with very limited power for clinical endpoints [9,10]. The aim of this study was a systematic review and meta-analysis of the effectiveness of local drug delivery with DEB (either in conjunction with bare-metal stenting or following plain old balloon angioplasty (POBA)) versus conventional treatment modalities in *de novo* coronary lesions.

## Methods

The study was performed according to the Preferred Reporting Items for Systematic Reviews and Meta-analyses (PRISMA) guidelines for meta-analyses of randomized trials (see Additional file 1) [11,12]. Planning and study design were done by two authors (GF, PM), including creation of an electronic database with variables of interest. Primary and secondary endpoints, variables of interest and search strategy (databases, sources for unpublished data) were defined in a strategy outline which can be obtained from the study authors on request. All studies included in this analysis were performed with the approval of an appropriate ethics committee and in compliance with the Helsinki Declaration.

### Search strategy

We searched EMBASE, PubMed, MEDLINE, BIOS and ISI Web of Science from 2005 through 28 December 2012. In addition, abstract lists and conference proceedings from the 2006 to 2012 scientific meetings of the American College of Cardiology, the European Society of Cardiology, the Symposium on Transcatheter Cardiovascular Therapeutics, the American Heart Association, and the World Congress of Cardiology were searched. We have not included studies published before 2005, as the main priority of this review is to illustrate the updated literature and the current effects of the different presented techniques on specific endpoints (for example, without significant effects of changes in clinical practice over time. We also considered published review articles, editorials and internet-based sources of information (http://www.tctmd.com, http://www.theheart.org, http://www.europcronline.com, http://www.cardiosource.com and http://www.crtonline.com) to assess potential information on studies of interest. Reference lists of selected articles were reviewed for other potentially relevant citations. No language restriction was applied.

The search terms used included "drug coated balloon", "drug eluting balloon", “randomized controlled trial”. The detailed search syntax for the database Medline is shown in Additional file 2. The syntax for other databases was similar but was adapted where necessary.

### Study selection

**Figure 1:**
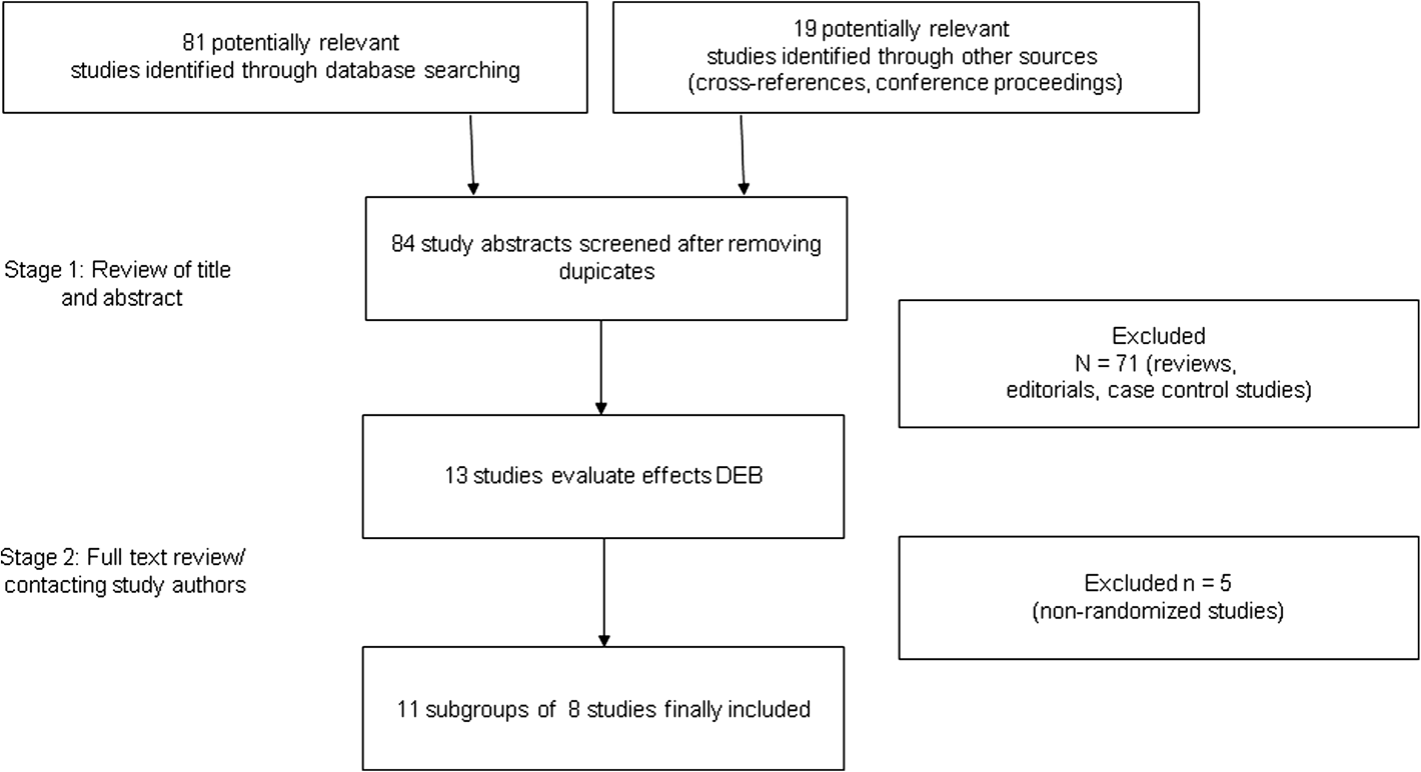
**Study selection process.** DEB drug eluting balloon.

In a two-step selection process, the titles and abstracts of all citations were reviewed by two researchers (PM, GF) to identify potentially relevant studies. In a second step, the corresponding publications were reviewed in full text to assess if studies met the following inclusion criteria: drug eluting balloon versus comparator treatment, randomized controlled trial (Figure 1).

### Data extraction and quality assessment

Relevant information from the articles including baseline clinical characteristics of the study population and outcome measures were extracted using the prepared standardized extraction database (Microsoft EXCEL). We assessed trial quality by evaluating randomization and allocation concealment, intention-to-treat analysis, blinded assessment of outcome measures, premature stopping of patient enrolment, and reporting on dropouts, but without using a quality score given the limitations inherent to such an approach (Table 1) [13].

**Table 1:** Quality assessment of studies investigating DEB + BMS vs control groups

**Study**	**Control group(s)**	**Randomization/ allocation concealment**	**Intention to treat analysis**	**Blinded endpoint assessment**	**Premature stopping**	**Reporting of dropouts**
DEB-AMI	DES, BMS	2	2	2	2	2
Stella *et al.*	DES, BMS	2	2	2	1	2
PEPCAD III	DES	1	2	0	1	1
PERFECT	EPC-BMS	2	2	1	1	1
BELLO	DES	2	2	1	1	2
Herdeg *et al.*	DES, BMS	2	2	1	2	2
Ali *et al.*	DES	2	1	1	1	1
PICCOLETTO	DES	2	2	1	2	2

Scoring: 0 points = no information or insufficient. 1 = Sufficient quality. 2 = Good quality.

### Endpoints and definitions

Baseline variables, and clinical and angiographic data were extracted. Variables of interest were a composite of major adverse cardiac events (MACE), target lesion revascularization (TLR), all-cause mortality, myocardial infarction, in-stent restenosis (≥50% diameter stenosis) and late lumen loss (LLL, in segment). For the definition in the individual trial, see Table 2.

**Table 2:** Baseline characteristics of included trials

**Study**	**Paclitaxel eluting balloon**	**Controls stent type(s)**	**Setting**	**Clopidogrel (mts)**	**Follow-up (mts)**	**Primary endpoint**	**MACE**	**TLR**	**Bare metal stenting**
DEB-AMI	DIOR 2^nd^ generation	TAXUS DES, Genius Magic Euroscore BMS	STEMI	12	6	LLL	death, MI, TVR	restenosis >50% ischemia	100%
Stella *et al.*	DIOR 1^st^ generation	TAXUS DES, Liberté BMS	stable/unstable CAD, bifurcation	3 after BMS, 12 after DES	12 (angio 6)	LLL	death, MI, TVR	restenosis >50% ischemia	100%
PEPCAD III	Coroflex DEBlue	Cypher DES	stable/unstable CAD	1 after DEB	9	LLL	NA	NA	100%
PERFECT	SeQuent Please+ PERFECT Stent	PERFECT Stent (EPC capturing Stent)	Stable CAD	3	6	LLL	death, MI, TLR	NA	100%
BELLO	IN.PACT Falcon	TAXUS DES	stable/unstable CAD small vessels	3 after DEB, 12 after DES	6	LLL	death, MI, TVR	any repeat revascularization	20.2%
Herdeg *et al.*	GENIE Acrostak	TAXUS DES, Multi-Link BMS	stable CAD	6	6	LLL	death, MI, TVR, stent thrombosis	any repeat revascularization	100%
Ali *et al.*	SeQuent Please	TAXUS DES	stable CAD in diabetics	NA	9	LLL	NA	NA	100%
PICCOLETTO	DIOR 1^st^ generation	TAXUS DES	stable/unstable CAD small vessels	1 after DEB, 3 after BMS, 12 after DES/unstable	9 (angio 6)	diameter stenosis	death, STEMI, TLR	>50% restenosis	NA (>100%)

CAD, coronary artery disease; DEB, drug eluting balloon; DES, drug eluting stent; EPC, endothelial progenitor cells; EPC-BMS Endothelial Progenitor Cell capturing Bare Metal Stent; LLL, in-stent Late Lumen Loss; MACE, definition of major adverse cardiac events; MI, myocardial infarction; mts, months; STEMI, ST-Elevation Myocardial Infarction; TLR, target lesion revascularization; TVR, target vessel revascularization.

### Data synthesis and analysis

Data of included studies were combined to estimate the pooled impact (risk ratio, RR) of DEB versus a comparator treatment. Study subgroups comparing DEB with either DES or BMS were the unit of analysis, DES and BMS are rather different comparators. Calculations were based on a DerSirmonian and Laird random-effects model [14]. This model assumes that the true effects vary between studies for unknown reasons. The primary summary measure usually reported is the estimated average effect across studies [15]. Continuity correction was used when no event occurred in one group to allow calculation of a RR [16]. Heterogeneity among trials was quantified with Higgins' and Thompson's I^2^[17]. *I*^*2*^ can be interpreted as the percentage of variability due to heterogeneity between studies rather than sampling error. An *I*^*2*^ >50% was considered as at least moderate heterogeneity. We present our primary result estimates of the average effect across studies with 95% confidence intervals in brackets. In addition, we also calculated 95% prediction intervals as described by Higgins *et al.*[15,18]. These intervals predict the effect that we would potentially expect to see in a new study. These data are presented in the sensitivity analysis paragraph. We did not test for publication bias or small study effects due to the small number of studies included in this analysis.

We performed stratified analyses for the different comparator treatments (BMS or DES). We performed sensitivity analyses excluding the one trial which was not yet published in full text but only as an abstract, [19] the trial which did use DEB alone (without BMS), [20] excluding the trial using progenitor cell capturing stents [21], and excluding the study using a special drug delivery catheter instead of a drug eluting balloon [22].

All analyses were performed with R, version 2.15.1 (package “meta”), R Foundation for Statistical Computing, Vienna, Austria [23].

## Results and discussion

### Description of included studies

A total of 90 articles were reviewed and 8 studies (11 subgroups) including 1,706 patients satisfied the predetermined inclusion criteria (Figure 1) [9,10,19-22,24,25]. Studies using DEB for in-stent restenosis treatment were not considered. All studies used paclitaxel-eluting balloons or delivery catheters. The DEB AMI trial enrolled only patients with STEMI while all the others excluded this patient group [10]. The eight studies presented seven subgroups where DEB was compared with a DES and four subgroups comparing DEB with BMS. Table 1 shows the baseline characteristics of the included studies.

### Major adverse cardiac events MACE

**Figure 2:**
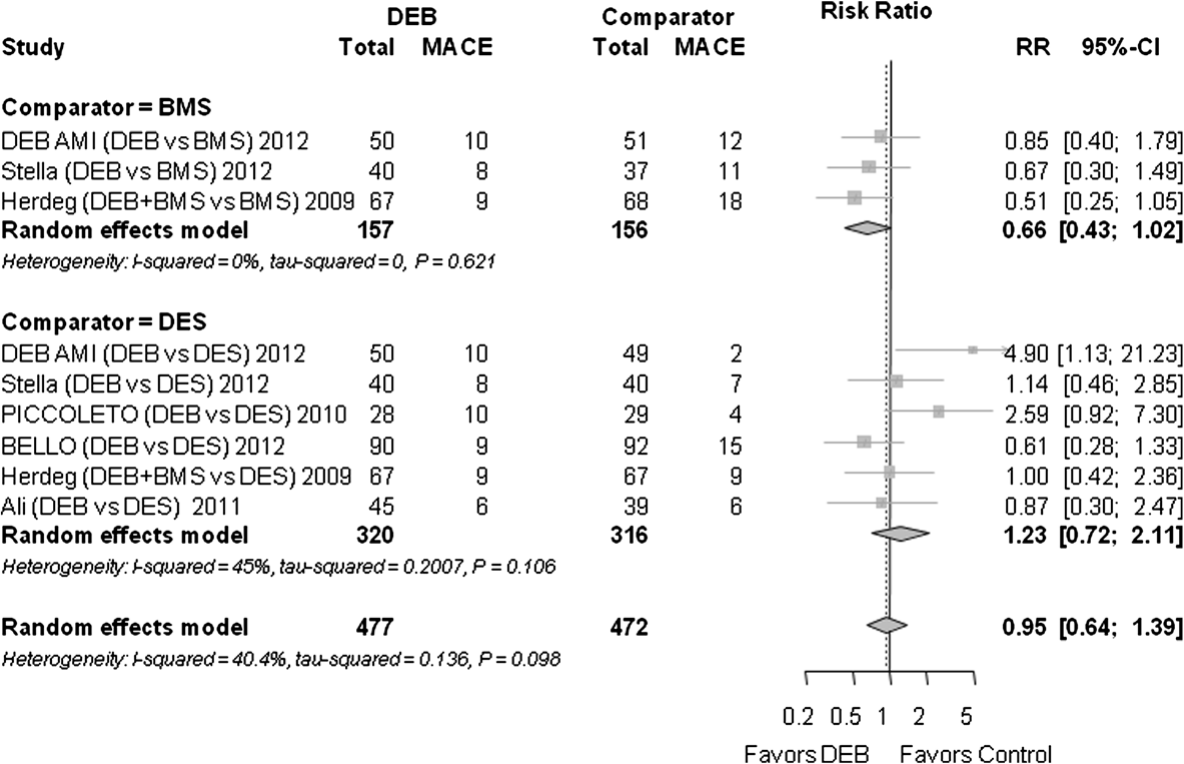
**Forest plot of risk ratios (RR) for major adverse cardiac events.** CI, confidence interval; DEB, drug eluting balloon; MACE, major adverse cardiac events; RR, risk ratio. Markers represent point estimates of risk ratios, marker size represents study weight in random-effects meta-analysis. Horizontal bars indicate 95% confidence intervals.

The definition of MACE differed slightly among the trials (Table 1). Overall, DEB were not superior to the control group (stenting with DES or BMS) (RR 0.95 (0.64 to 1.39); *P* = 0.776) (Figure 2).

### Target lesion revascularization

**Figure 3:**
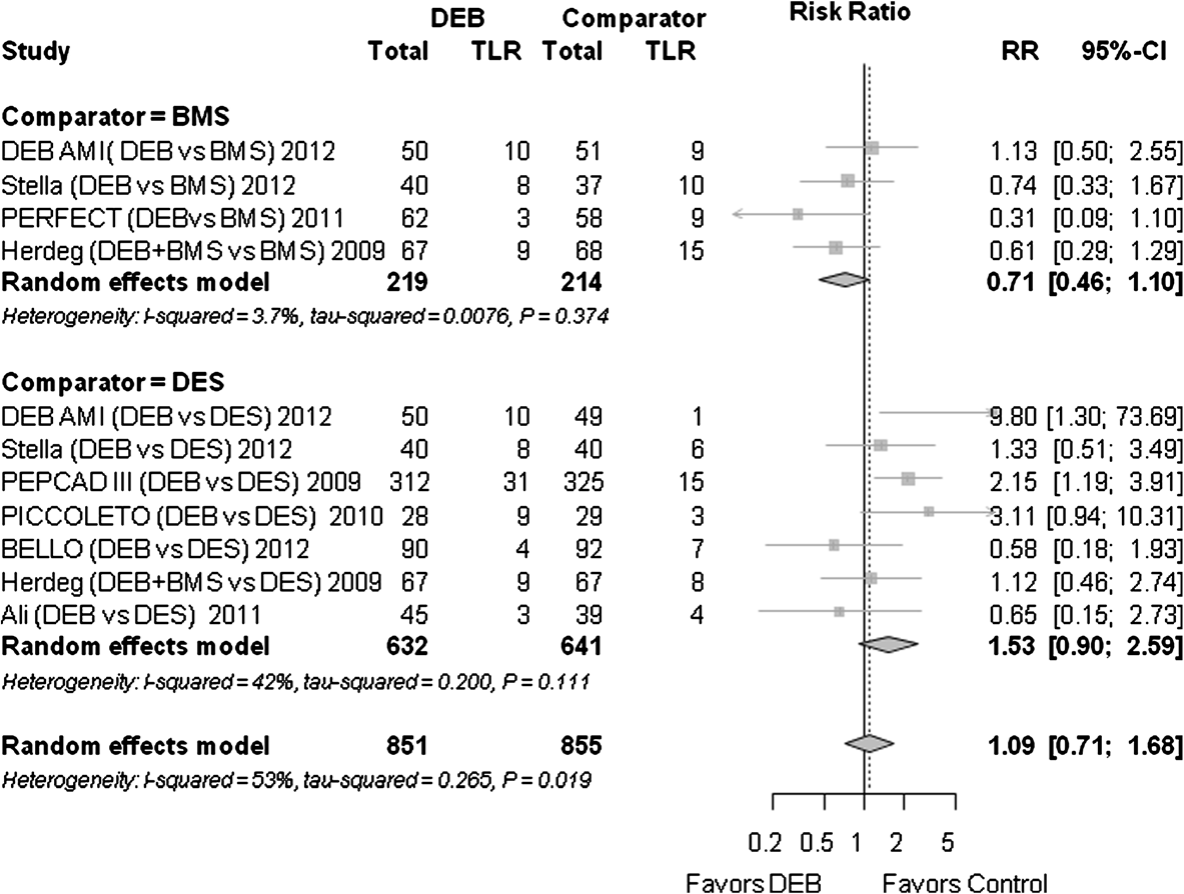
**Forest plot of risk ratios (RR) for target lesion revascularization.** CI, confidence interval; DEB, drug eluting balloon; RR, risk ratio; TLR, target lesion revascularization.

The need for target lesion revascularization (TLR) was not significantly different between DEB and the control group (1.09 (0.71 to 1.68); *P* = 0.700). (Figure 3) In most studies, TLR was clinically driven (Table 1).

### Binary in-segment restenosis

**Figure 4:**
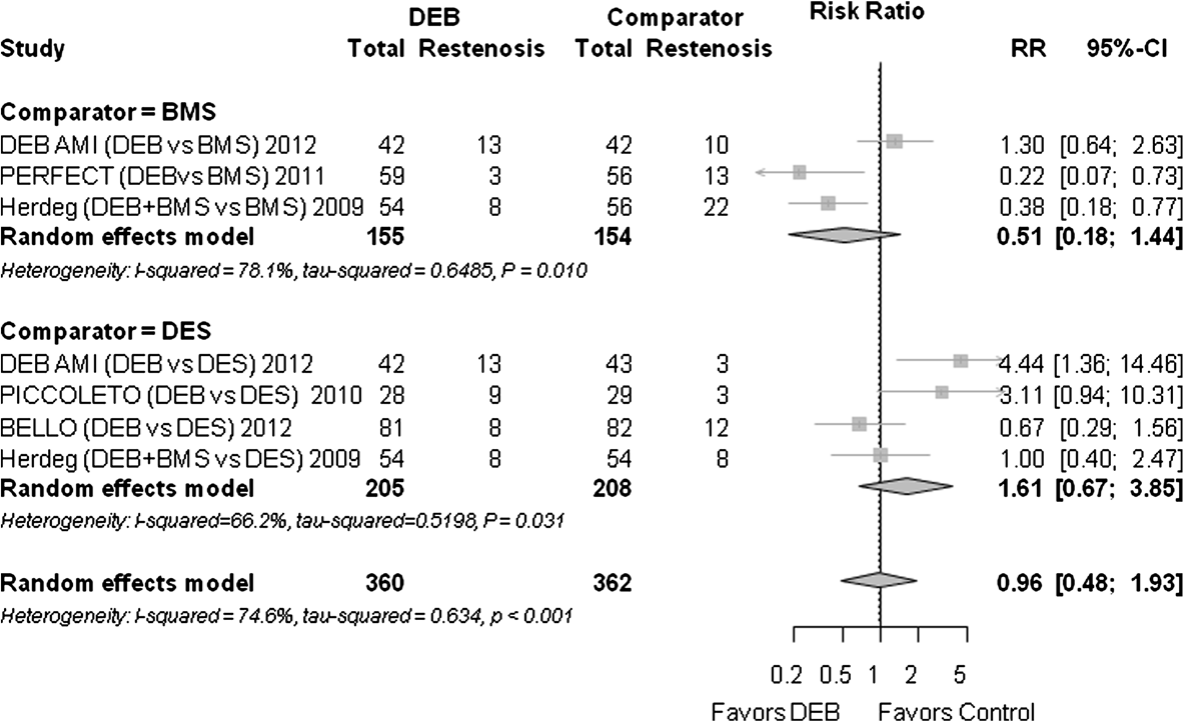
**Forest plot of risk ratios (RR) for restenosis (≥50% diameter-stenosis).** CI, confidence interval; DEB, drug eluting balloon; RR, risk ratio.

The rate of in-segment restenosis was similar between DEB and the control group (0.96 (0.48 to 1.93), *P* = 0.918) (Figure 4).

### Late luminal loss

**Figure 5:**
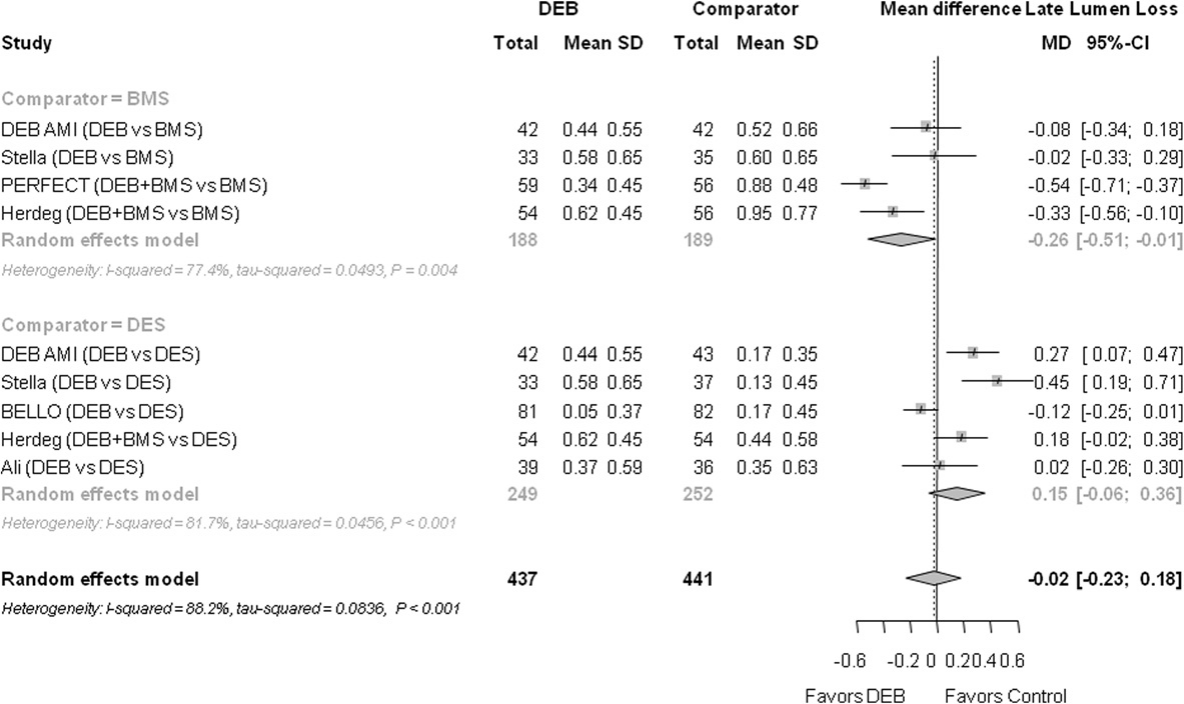
**Forest plot of risk ratios (RR) for late lumen loss.** CI, confidence interval; DEB, drug eluting balloon; SD, standard deviation.

The LLL was similar for DEB and the control group (mean difference - -0.02 mm (−0.23 to 0.18); *P* = 0.818) (Figure 5).

### All-cause mortality

**Figure 6:**
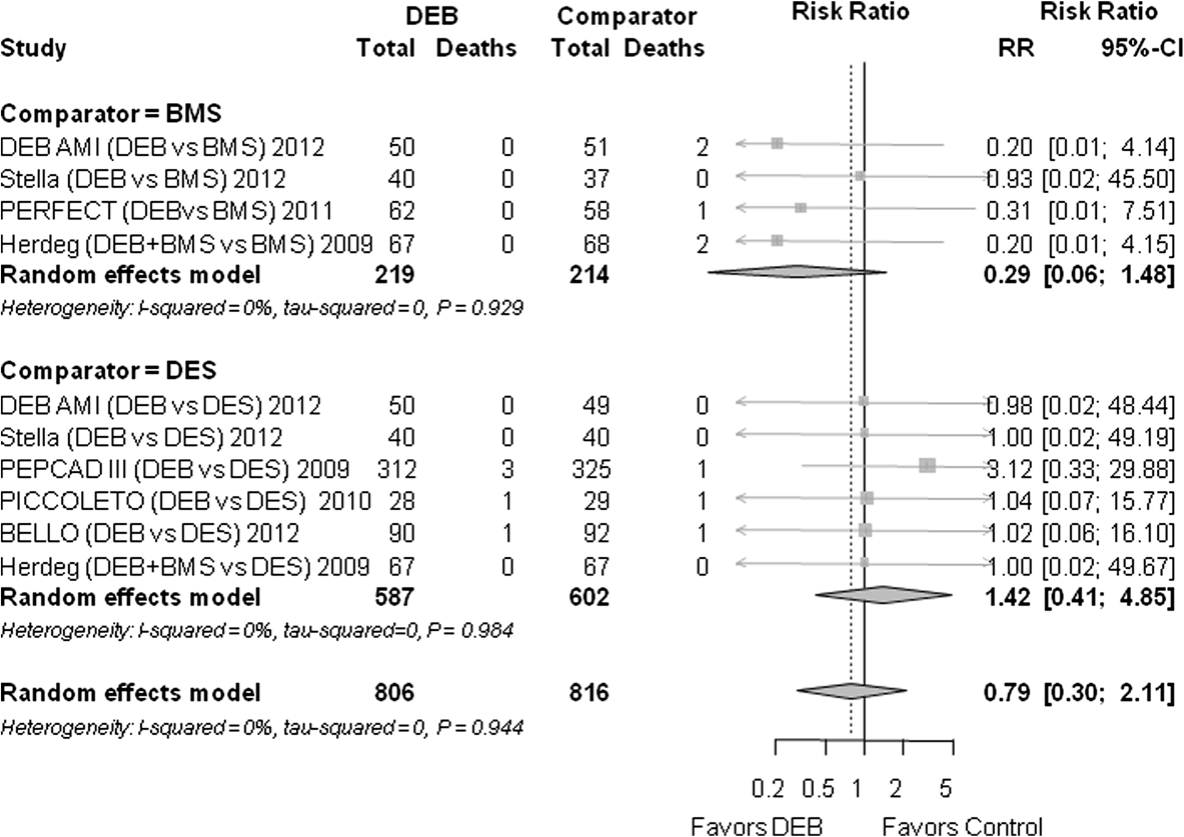
**Forest plot of risk ratios (RR) for mortality.** CI, confidence interval; DEB, drug eluting balloon; SD, standard deviation.

The mortality was not significantly different for DEB and the control group (0.79 (0.30 to 2.11), *P* = 0.644) (Figure 6).

### Myocardial infarction

**Figure 7:**
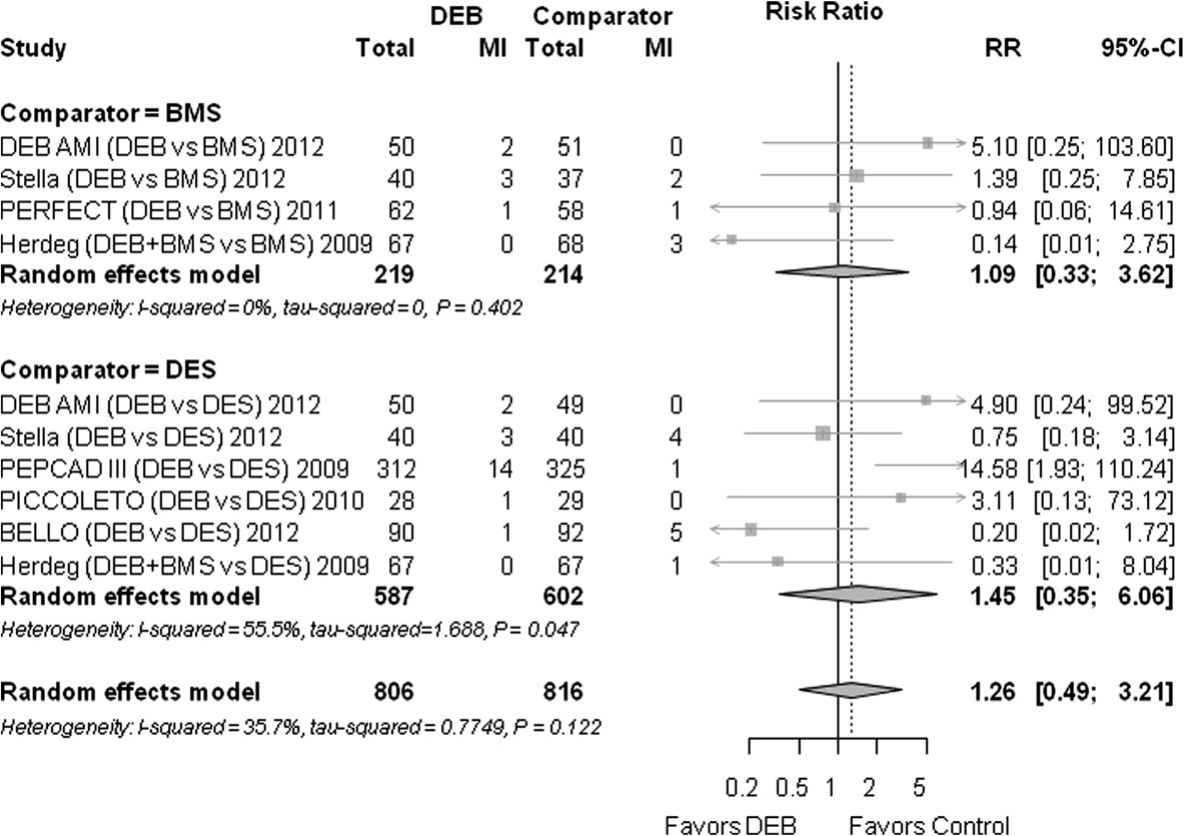
**Forest plot of risk ratios (RR) for myocardial infarction.** CI, confidence interval; DEB, drug eluting balloon; MI, myocardial infarction; SD, standard deviation.

Overall, the risk for myocardial infarction was not significantly different between DEB and the control group (RR 1.26 (0.49 to 3.21), *P* = 0.629) (Figure 7).

### Stent thrombosis

**Figure 8:**
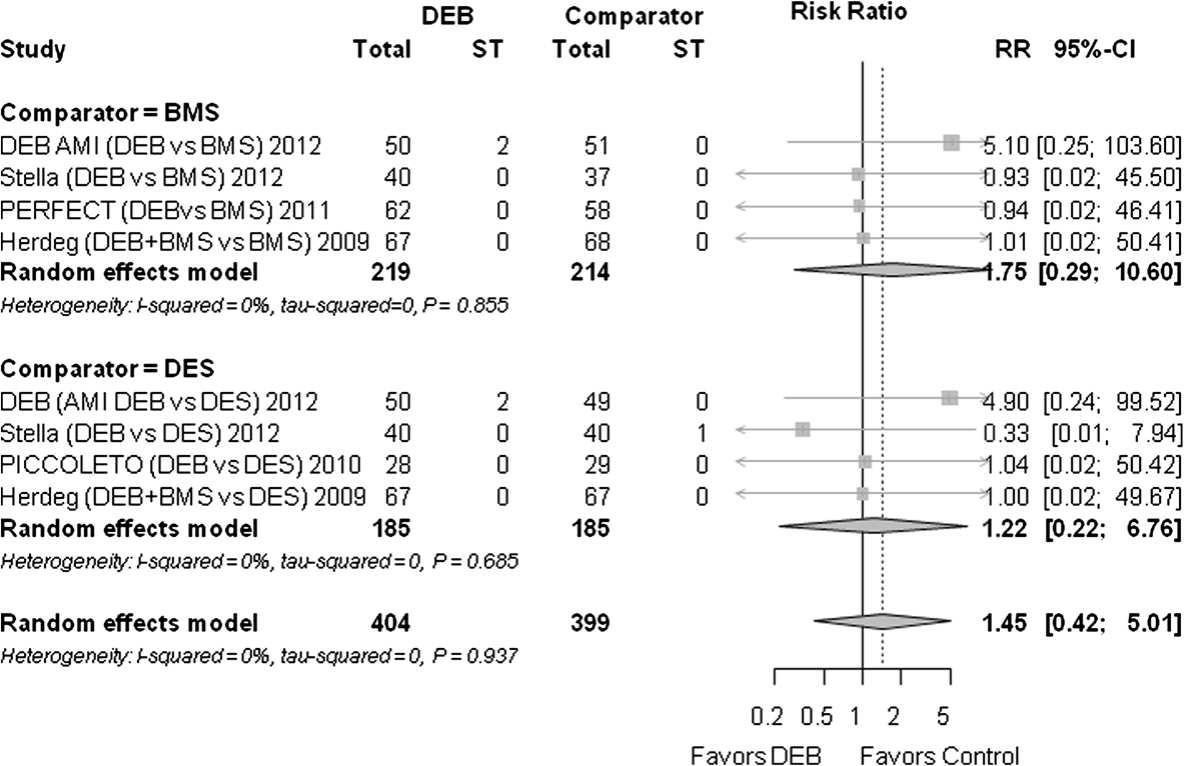
**Forest plot of risk ratios (RR) for stent thrombosis.** CI, confidence interval; DEB, drug eluting balloon; SD, standard deviation; ST, stent thrombosis.

Overall, stent thrombosis (ST) was a very rare event and not significantly different between DEB and the control group (RR 1.45 (0.42 to 5.01), *P* = 0.560) (Figure 8).

### Sensitivity analyses

We also calculated the prediction intervals for those clinical endpoints that were statistically significant. These intervals predict the effect that we would potentially expect to see in a future study. The prediction intervals all crossed the line of unity and were, therefore, not significant. The influence analyses, omitting one trial at a time, showed rather robust results that were not relevantly influenced by a single trial (Table 3).

**Table 3:** Sensitivity analyses

**Endpoint**	**Confidence intervals**	**Prediction intervals**	**Only published data**	**Without STEMI**	**Only combined DEB+BMS**	**Excluding progenitor cell stent**	**DEB exclusively**
MACE	0.95 (0.64 to 1.39)	0.95 (0.35 to 2.52)	0.95 (0.64 to 1.39)	0.84 (0.61 to 1.15)	0.84 (0.61 to 1.15)	0.95 (0.64 to 1.39)	1.07 (0.67 to 1.70)
TLR	1.09 (0.71 to 1.68)	1.09 (0.31 to 3.83)	0.97 (0.63 to 1.50)	1.01 (0.68 to 1.51)	1.00 (0.65 to 1.55)	1.20 (0.79 to 1.82)	1.18 (0.70 to 1.99)
Binary restenosis	0.96 (0.48 to 1.93)	0.96 (0.10 to 9.05)	0.96 (0.48 to 1.93)	0.77 (0.40 to 1.48)	0.82 (0.40 to 1.66)	1.18 (0.59 to 2.35)	1.20 (0.48 to 2.96)
MI	1.26 (0.49 to 3.21)	1.26 (0.13 to 11.83)	0.86 (0.40 to 1.85)	1.13 (0.42 to 3.06)	1.18 (0.43 to 3.24)	1.30 (0.46 to 3.65)	1.69 (0.62 to 4.59)
Mortality	0.79 (0.30 to 2.11)	NA	0.58 (0.20 to 1.71)	0.78 (0.29 to 2.15)	0.76 (0.27 to 2.18)	0.88 (0.31 to 2.45)	0.93 (0.32 to 2.71)
ST	1.45 (0.42 to 5.01)	NA	1.45 (0.42 to 5.01)	1.13 (0.29 to 4.40)	1.50 (0.41 to 5.57)	1.52 (0.41 to 5.63)	1.59 (0.40 to 6.36)

BMS, bare metal stent; DEB, drug eluting balloon; MACE, major adverse cardiac events; MI, myocardial infarction; RR, relative risk (and 95% confidence intervals); ST, stent thrombosis; TLR, target lesion revascularization.

### Subset analyses

We have also analyzed the data for the subset of trials which compared DEB with drug eluting stents or with bare metal stents.

#### MACE

Compared to BMS, there was a trend towards a lower MACE risk for DEB (RR 0.66 (0.43 to 1.02), *P* = 0.060). Compared to DES, there was no significant difference (RR 1.23 (0.72 to 2.11), *P* = 0.750).

#### TLR

Compared to BMS, the TLR for DEB was not significantly different (RR 0.71 (0.46 to 1.10), *P* = 0.120). Compared to DES, the RR was 1.53 (0.90 to 2.56); *P* = 0.117.

#### Binary restenosis

Compared to BMS, the RR was 0.51 (0.18 to 1.44), *P* = 0.203. Compared to DES, the RR was 1.61 (0.67 to 3.85), *P* = 0.286.

#### Late lumen loss

Compared to BMS, LLL was lower for DEB (− 0.26 mm (−0.51 to −0.01); *P* = 0.040). Compared to DES, LLL was not significantly different (0.15 mm (−0.06 to 0.36); *P* = 0.162).

#### Mortality

Compared to BMS, the RR for DEB was 0.29 (0.06 to 1.48), *P* = 0.137.

Compared to DES the RR was 1.42 (0.41 to 4.85), *P* = 0.579.

#### Myocardial infarction

Compared to BMS, the RR was 1.09 (0.33 to 3.62); *P* = 0.893.

Compared to DES, the RR was 1.45 (0.35 to 6.06); *P* = 0.613.

#### Stent thrombosis

When compared to BMS the RR was 1.75 (0.29 to 10.60), *P* = 0.545, compared to DES it was 1.22 (0.22 to 6.76), *P* = 0.819.

We have also analyzed the data of the two trials which did compare DEB alone (and only provisional stenting with a bare metal stent) versus DES (BELLO+PICOLETTO). For MACE the RR was 1.21 (0.30 to 4.94); *P* = 0.793. For mortality it was 1.03 (0.148 to 7.14); *P* = 0.977. For MI the RR was 0.61 (0.05 to 8.41); *P* = 0.715. The RR for TLR was 1.35 (0.26 to 6.92); *P* = 0.722 and for binary re-stenosis the RR was 1.36 (0.31 to 6.06); *P* = 0.686.

## Discussion

This is the first meta-analysis assessing the clinical effectiveness of drug-eluting balloons to treat *de novo* coronary lesions. Overall, DEB were not superior to current standard therapy using DES or BMS. However, the present study shows signals suggesting that the use of DEB may be superior to BMS alone although larger trials are needed for confirmation. DEB may be beneficial in patients who are not eligible for DES implantation or in small vessel disease, where stenting is undesirable or not possible.

DEB are an established treatment for in-stent restenosis and recommended by current European guidelines (class IIa, level of evidence B) [26]. The role of DEB for percutaneous coronary intervention (PCI) of *de novo* coronary lesions has not been well defined yet. Currently, neither the European nor American guidelines recommend DEB for *de novo* lesions.

### Potential benefits of DEB for treatment of *de novo* coronary lesions

DES implantation is the gold standard for percutaneous treatment of *de novo* lesions in major coronary vessels [26]. However, there are several pitfalls related to DES use where DEBcould find its niche as a potent DES alternative:

1) After DES implantation, dual antiplatelet therapy is mandatory for 6 to 12 months [26]. Therefore, patients who are at an increased bleeding risk or who are awaiting urgent surgery will mainly receive BMS, where dual antiplatelet therapy is required for one month only. Notably, implantation of BMS is associated with an elevated risk for in-stent restenosis (ISR) of 20 to 30% compared to 10 to 15% with DES after one year [26,27]. In diabetics, representing a high risk population, the relative risk for ISR is further doubled with BMS [28]. In contrast, local drug delivery via a DEB and consecutive implantation of a BMS might result in both, a reduced rate of ISR and a significantly reduced duration of dual anti-platelet therapy if compared to DES. In particular, clopidogrel is recommended for one to three months after DEB-PTCA (percutaneous transluminal coronary angioplasty), at least in the setting of PCI for stable CAD [19,24]. In the setting of small vessel disease, where the results of balloon angioplasty appear non-inferior to those after stenting, the use of DEB only might be a reasonable option [29].

2) An appropriate technique of drug delivery to prevent ISR is crucial for a favorable long-term outcome [30,31]. Not only the optimal stent/balloon design (for example, design and diameter of the stent struts), but also the properties of the ideal carrier matrix are hotly debated [32]. Among different DES platforms, several authors have been able to demonstrate an association of (late) stent thrombosis with the polymer and ISR due to a delayed healing and endothelialization process [33,34]. More recently, bioabsorbable polymers that leave, in effect, a BMS after drug delivery have been developed with promising results [35]. Even polymer-free DES, with less reliable drug-release-kinetics, have been proposed to overcome the burden of depleted permanent polymers [36]. However, all of these proposed DES types require antiplatelet therapy for at least six months [27].

3) DEB deliver higher paclitaxel doses (300 to 600 ug with DEB vs. 100 to 200 ug with DES), and as the drug eluting stent struts commonly cover only 20% of the injured vessel wall, the larger DEB surface area guarantees more uniform drug delivery [37].

### Current evidence for DEB treatment in *de novo* coronary lesions

In the present study, we investigated eight randomized controlled trials, involving 1,706 patients, who underwent DEB and bare metal stenting versus implantation of conventional BMS or DES for *de novo* coronary lesions. Overall, there were no significant differences between the DEB and the control groups in terms of mortality or MACE.

In particular, there appears to be a trend towards a beneficial effect of DEB if compared to BMS alone. Contrarily, DEB tended to be inferior compared to DES. Therefore, patients who are eligible for a DES should currently not be treated with a DEB. However, DEB might be considered as a valid option for patients who are at high risk for in-stent restenosis and those who cannot receive a DES.

The possible reasons for the inferior performance of DEB in comparison to DES remain speculative, especially as all balloons and the vast majority of DES in the analysis were covered with paclitaxel. Balloons were covered with (1-)3 ug/m2 paclitaxel [37], whereas the predominantly used DES, TAXUS Liberte slow release stent, incorporates 1 ug/mm2. As outlined above, not only stent design and the anti-proliferative drug, but also the release kinetics from the balloon/stent surface into the vessel wall are crucial. Indeed, the drug release kinetics of DEB are very different from DES and might explain the divergent results. Following POBA with a standard balloon, DEB are inflated for 30 to 120 seconds, in accordance with the manufacturers’ recommendations. Despite this relatively short contact time between balloon and vessel wall and the rapid decay of paclitaxel blood concentration, paclitaxel penetrates the vessel wall easily due to its highly lipophilic properties. Furthermore, the biologic effects of paclitaxel last for several days and although in some DEB there is an excipient to facilitate drug transfer there is no permanent polymer involved ([37]). In contrast, the TAXUS Liberté stent releases paclitaxel very slowly and the carrier polymer is permanently attached to the stent platform [37].

In several long-term, follow-up trials the TAXUS stent was associated with an increased risk of late stent thrombosis, occurring sometimes even more than two years after the index intervention [27]. A potential impact of DEB use on the rate of late stent thrombosis might have been undetected in the present meta-analysis as the average follow-up period of eight months was relatively short. Of note, the reported number of in-stent thromboses was very low in all studies.

It has to be considered that different types of drug-eluting balloons have been used among trials included in this meta-analysis (Table 2) The PICCOLETO trial, for example, used the first generation Dior balloon. This balloon has been iterated and used a new carrier (Shellac®) which demonstrated an about 20-fold increased paclitaxel concentration in the tissue [38]. This progress in DEB technology may lead to improved results in future trials.

### Limitations

This meta-analysis is only based on relatively small and very heterogeneous randomized controlled trials. Although the formal testing did not reveal a major inter-study heterogeneity, there are major differences between the study designs. The main comparators were the DES or BMS. These stent platforms perform rather differently and we, therefore, focused on stratified analyses for the comparator stent type (DES or BMS) and study subgroups as our unit of analysis. However, even the pooled analysis of the eight trials has a limited statistical power when stratifying the analysis based upon the stent type. This is especially the case for rare events such as stent thrombosis and death. These results have to be interpreted with caution. Apart from two studies (which used sirolimus [19] and an EPC capturing stent [22] respectively), paclitaxel eluting stents served as the DES control group in the investigated trials. Newer generation DES might, therefore, even outperform the results of the paclitaxel eluting stent in the present study.

Hard endpoint outcomes (myocardial infarction, definite or probable stent thrombosis and death) need to be interpreted with care since the meta-analysis might be too small to detect statistical differences in such rare events. Nevertheless, we find it reassuring that there appear to be no significant differences between DEB and BMS or DES from a safety standpoint.

### Outlook

Currently, paclitaxel is the only available anti-proliferative drug that is mounted onto the DEB. Different alternatives, like zotarolimus, sirolimus or everolimus, which have already been used successfully on different DES platforms, might also translate into improved outcomes when used for DEB [39]. Indeed, a zotarolimus eluting balloon has already been tested in a swine model [40]. The first DEB study is currently recruiting patients with stable CAD and will compare DEB/BMS vs. a zotarolimus eluting balloon (NCT01539603). Further studies are recruiting patients with STEMI (DEB/BMS vs. TAXUS NCT00856765) or NSTEMI (PEPCAD NSTEMI NCT01489449). The BASKET-SMALL2 trial will investigate the cost-effectiveness of DEB vs. TAXUS in small vessels (NCT01574534). Many more prospective randomized trials are currently recruiting patients to investigate the optimal use of DEB in *de novo* coronary lesions.

## Conclusion

Overall, the results of drug-eluting balloons (in combination with BMS or as stand-alone therapy) appear comparable to BMS and paclitaxel eluting stents for *de novo* coronary artery lesions. Compared to BMS exclusively, there are signals for a potential benefit of DEB, but larger trials would be needed to detect a significant difference. DEB may be beneficial in cases where bare metal stenting is considered as the only choice and patients are at high risk for ISR (for example, diabetics), to reduce the need for long term dual antiplatelet therapy and following POBA in small vessels. The heterogeneity between the included studies is a limitation of this meta-analysis; different drug-eluting balloons have been used.

## Abbreviations

BMS: Bare metal stent; CAD: Coronary artery disease; DEB: Drug eluting balloon; DES: Drug eluting stent; ISR: In-stent restenosis; LLL: Late lumen loss; MACE: Major adverse cardiac events; MI: Myocardial infarction; PCI: Percutaneous coronary interventions; POBA: Plain old balloon angioplasty; PRISMA: Preferred Reporting Items for Systematic Reviews and Meta-analyses; PTCA: Percutaneous transluminal coronary angioplasty; RR: Risk ratio; ST: Stent thrombosis; TLR: Target lesion revascularization.

## Competing interests

The authors declare that they have no competing interests.

## Authors’ contributions

GMF contributed to the study design, the literature search, data extraction, data interpretation, drafting of the manuscript, and provided critical revision of the manuscript for intellectual content. AJL, DTK, RDP and AT contributed to data interpretation and provided critical revision of the manuscript for intellectual content. OA contributed to data interpretation, statistical analysis and data extraction and provided critical revision of the manuscript for intellectual content. PM contributed to study design, the literature search, data extraction, statistical analysis and data interpretation and provided critical revision of the manuscript for intellectual content. All authors read and approved the final manuscript.

## Authors’ information

GMF is a fellow in interventional cardiology. Originally from Austria, he has trained in world-leading centers in Germany and Switzerland before coming to the UK. AJL is a cardiologist and an Associate Professor at Yale University and a renowned expert in clinical research in the field of invasive cardiology and leads the Yale-UCL Cardiovascular Research collaborative. DTK is an associate professor at the Institute for Clinical Evaluative Sciences, Toronto, and a world-expert in population-based and outcomes research as well as in meta-analyses. He has published his project very successfully in high impact scientific journals. OA has a master’s degree in public health and has a special interest in cardiovascular outcomes research and statistical methods in meta-analyses. RP is an interventional cardiology fellow and co-editor of one of the main text books on interventional cardiology, *The PCR-EAPCI Percutaneous Interventional Cardiovascular Medicine Textbook*. AT is a Professor of Cardiology and works as an interventional cardiologist in one of the highest volume cardiovascular centers in London, the London Chest Hospital. He is editor-in-chief of the journal *Heart*. PM is an interventional Cardiologist at University College London Hospitals UCLH, London, and part of the Yale-UCL Cardiovascular Research collaborative. For more information, see http://www.drpascalmeier.com).

## Pre-publication history

The pre-publication history for this paper can be accessed here:

http://www.biomedcentral.com/1741-7015/11/123/prepub

## Supplementary Material

Additional file 1 PRISMA 2009 Checklist. Click here for file

Additional file 2 Search strategy for MEDLINE (search date 13 December 2012). Click here for file
