# Supplementary material for: Drug eluting balloons for de novo coronary lesions – a systematic review and meta-analysis
Source: BMC Med. 2013 May 8;11:123. doi: 10.1186/1741-7015-11-123 (PMC3648374; doi:10.1186/1741-7015-11-123)
Supplement: Additional file 2 — Search strategy for MEDLINE (search date 13 December 2012). [file 1741-7015-11-123-S2.doc]

| Search | # of abstracts |
| --- | --- |

Search History

Limits: Humans, Randomized Controlled Trial, 81

#5 Search #1 OR #2 AND #3 AND #4 AND #5 1285

## #4 Search " Angioplasty, Balloon, Coronary"[Mesh] 21607

#3 Search "coronary stenosis"[Mesh] 3693

#2 Search "drug eluting balloon"[All] 4127

#1 Search "drug coated balloon"[All] 537

Supplementary File 2: Search strategy for MEDLINE (search date December 31, 2012).
